# The Overload Effect on the Crack Tip Damage Mechanism in a 7075 Aluminum Alloy

**DOI:** 10.3390/ma17164088

**Published:** 2024-08-17

**Authors:** Changji Xie, Zhanguang Zheng, Li Li, Teng Sun

**Affiliations:** 1 College of Mechanical Engineering, Guangxi University, No. 100 Daxue Dong Road, Nanning 530004, China; 19973297451@163.com; 2 Key Laboratory of Disaster Prevention and Structural Safety of Ministry of Education, Guangxi Key Laboratory of Disaster Prevention and Engineering Safety, Guangxi University, No. 100 Daxue Dong Road, Nanning 530004, China; 3 Beibu Gulf Key Laboratory of Ocean Engineering Equipment and Technology, Guangxi Key Laboratory of Ocean Engineering Equipment and Technology, College of Mechanical and Marine Engineering, Beibu Gulf University, Qinzhou 535011, China; suntengqz@163.com

**Keywords:** 7075 aluminum alloy, overload effect, crystal plasticity, fatigue damage

## Abstract

In the serviced components of a 7075 aluminum alloy, the propagation of fatigue crack can be retarded because of the overload effect; however, the corresponding retardation mechanisms are complex. To provide further insights into the retardation mechanisms of 7075 aluminum alloys, this study addresses the crack tip damage response of a cracked 7075 aluminum alloy under an overload effect. Based on the dual-scale modeling approach and the damage-coupled crystal plasticity model, the effect of the microstructure of a 7075 aluminum alloy on the damage behavior ahead of the crack tip under an overload was studied. The factors affecting fatigue damage accumulation ahead of the crack tip, such as dislocation density, the variation in the activities of slip systems, and the orientation effect of the nearest neighbor grains, are described. The results show that for the 7075 aluminum alloy, the compressive residual stress induced by the overload effect not only decreases the number of activated slip systems, but also lowers the rate of increase in dislocation density. This causes a decrease in fatigue damage accumulation during deformation. Moreover, the overload effect decreases the slip system activity as well as the resultant plastic slip; however, the decrease in plastic slip varies with the grain orientation, indicating that the overload effect depends on the grain orientation. It can also be found that both the damage strain energy release rate and lattice strain are influenced by the orientation of the nearest neighbor grains, which can eventually affect the overload effect. These findings contribute to understanding the retardation mechanisms from a microscopic perspective and provide guidance on improving the material design of a 7075 aluminum alloy to some extent.

## 1. Introduction

Components in service are generally subjected to variable amplitude cyclic loadings such as tensile overloads [1,2,3]. For aircraft, harsh weather, turbulence and hard landings can all cause tensile overloads [4,5]. A tensile overload can cause many materials to develop significant fatigue crack growth retardation [6,7,8,9], which indicates that the tensile overload effect can prolong fatigue life. A 7075 aluminum alloy is an excellent material that plays an important role in many industries. Like many other materials, its fatigue crack growth rate decreases after overload [10,11,12]. In this study, the retardation mechanism of a 7075 aluminum alloy is investigated.

Studies on the tensile overload effect date back to the 1960s [6]. Plasticity-induced crack closure [13,14,15], together with compressive residual stress [16,17,18], is widely used for the analysis of the overload effect. Crack branching [19], crack tip blunting [20,21], and strain hardening [22] can also cause the retardation of fatigue crack growth. Apart from these mechanisms, the stress and plastic strain fields and the damage accumulation ahead of the crack tip have also been studied to understand crack behavior under cyclic loading with overload [23,24,25,26,27]. Xu et al. [23] found that the overload effect reduced the stress at the moment when the peak value of loading was applied. This finding implied that the effective stress decreased due to the presence of the overload effect. Jiang et al. [24] revealed that inhomogeneous plastic deformation, depending on the overload, induced compressive residual stress. Toribio and Kharin [25] investigated the near-tip accumulative plastic strain and revealed that the rate of increase in accumulative plastic strain was altered after overload. Moreover, Toribio and Kharin [26] found that the stress and strain fields ahead of the crack tip were correlated to the retardation caused by the overload effect because they governed damage accumulation and consequently fatigue cracking. Bahloul and Bouraoui [27] reported that the ratcheting strain as an indicator of the damage degree tended to decrease after overload due to the retardation effect. The studies mentioned above explain the retardation mechanisms from a macroscopic perspective. However, polycrystalline materials consist of grains with different morphologies and crystallographic orientations [28,29], which are responsible for the complex and inhomogeneous mechanical behavior of polycrystalline materials [29]. In other words, the fatigue crack behavior is greatly influenced by the grain microstructure [30]. Therefore, to further understand the retardation mechanisms of a 7075 aluminum alloy, the response ahead of the crack tip under the cyclic load with a single overload needs to be investigated from the microscopic perspective.

Numerous researchers have been motivated to investigate the relationship between the grain microstructure of materials and the overload retardation in recent years. Their studies focus on lattice strain [31,32], crystal plastic deformation distribution [30,31,33], crystallographic orientation variation [31,33], and the grain size effect [34,35]. Ahead of the crack tip, plastic deformation and a high variation in crystallographic orientation are caused by the overload. Crystal plastic deformation acts as a high energy barrier and is able to prevent the expansion of the fatigue crack [31]. For the grains surrounding the crack tip, their local crystallographic orientations become identical because of the high variation in crystallographic orientation, which reduces the driving force of intergranular crack propagation [31]. Grain size is a critical factor influencing crystal plastic deformation under overload. The smaller grains have higher strength and are more difficult to deform, which causes the plastic zone at the crack tip to become smaller [34]. In addition, the compressive residual stress resulting from the overload effect acts on the plastic zone generated near the crack tip and affects the development of the lattice strain [31,32,36]. Lam et al. [31] studied the CoCrFeMnNi high-entropy alloy under overload and found that the conspicuous deformation twins appeared within the (111)-oriented grains along the crack path. By using the electron backscatter diffraction, they also observed that only the (001) orientation concentrated around the crack tip, inhibiting the fatigue crack propagation after overload. Zhang et al. [34] found that under the same overload condition, coarse-grained nickel had the highest fatigue crack propagation rate after overload, followed by ultrafine-grained nickel, and nanocrystalline nickel had the lowest fatigue crack propagation rate. Lee et al. [32] studied the lattice strain at the crack tip of a nickel-based HASTELLOYC-2000 alloy and revealed that lattice strain evolution was influenced by the compressive residual stress that arose from the overload effect. Refs. [31,32,34] show that for face centered cubic (FCC) materials, overloading causes a variation in lattice strain and crystal plastic deformation; moreover, grain size can affect the overload effect. It can be extrapolated that these conclusions are also suitable for a 7075 aluminum alloy because this alloy is categorized as an FCC material. However, cyclic loading with overload causes damage accumulation, and it should be noted that the grains differ in fatigue damage evolution because of their different crystallographic orientations [37,38]. Yuan et al. [38] revealed the mechanisms of fatigue damage based on a simulation conducted using their own crystal plasticity model. They reported that crystallographic orientation influenced local stress distribution, and this local stress distribution determined the different locations of fatigue crack initiation. This indicates that crystallographic orientation is correlated to fatigue damage. Moreover, the fatigue damage of grains is closely associated not only with dislocation density [39,40], but also with interactions between neighbor grains [41]. The interactions between neighbor grains also have a significant effect on lattice strain because lattice strain is caused by the deformation incompatibility of neighbor grains [42]. Therefore, this study takes these factors into account with the goal of providing further insights into the retardation mechanism of a 7075 aluminum alloy under overload.

To achieve this goal, the response ahead of the crack tip of a 7075 aluminum alloy under overload was simulated based on a dual-scale modeling approach and two fatigue-coupled constitutive models. The Chaboche model and the crystal plasticity finite element model were coupled with the same low-cycle fatigue damage model. The former and the latter were applied to the macro-scale and micro-scale models, respectively. The crystal plasticity model describes the response of each grain based on the plastic slip occurring in the slip systems [43,44]. The outline of this study is as follows: Section 2 describes the dual-scale model, and constitutive models are presented in Section 3. Subsequently, Section 4 discusses damage accumulation under overload and its relevant influencing factors. Finally, the conclusions are summarized in Section 5.

## 2. Dual-Scale Modeling

**Figure 1:**
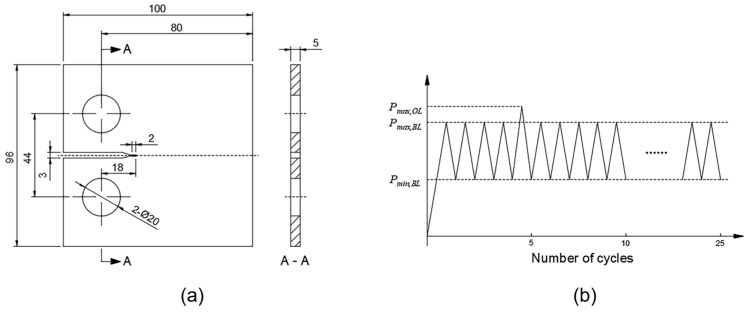
(**a**) Dimensions of the CT specimen; (**b**) cyclic loading with a single tensile overload.

A dual-scale modeling approach is employed here to save on computational costs. The macro-scale model is established based on the compact tensile (CT) specimen, and its role is to provide the micro-scale model with boundary conditions. Figure 1a,b shows the dimensions of the macro-scale model and the cyclic loading condition, respectively. The peak value of the baseline load is 12.5 kN, and the load ratio of the baseline load is set as 0.5. According to the study by Lu et al. [45], the overload ratio (*R*_OL_) is the ratio of the overload value to the peak value of the baseline load. Moreover, two overload ratios of 1.1 and 1.2 are used here.

**Figure 2:**
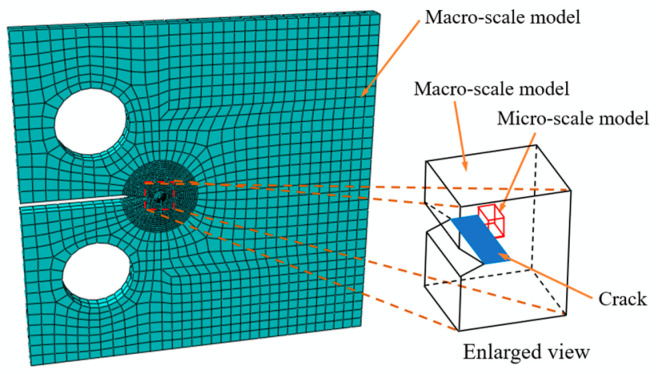
Schematic diagram for the location of the micro-scale model.

**Figure 3:**
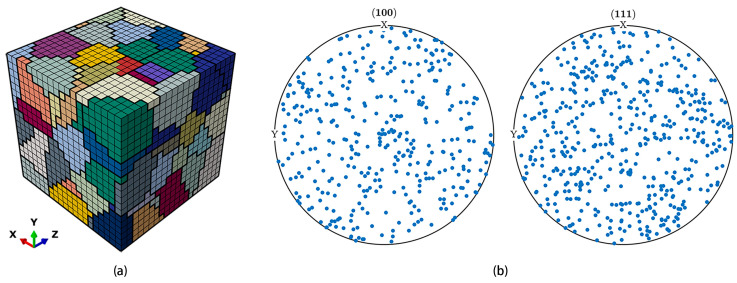
(**a**) RVE model consisting of 125 grains; (**b**) pole figures of <100> and <111>.

The micro-scale model for microscopic analysis can be obtained by using the partition of the macro-scale model. As shown in Figure 2, the micro-scale model is located ahead of the crack tip. A representative volume element (RVE) model containing 125 grains, shown in Figure 3a, is acquired by using the Voronoi tessellation technique and is a cube whose side length is 0.5 mm. It is adopted here to represent the micro-scale model. Each individual grain has a random shape, size, and crystallographic orientation. The crystallographic orientation distribution of the 125 grains is shown in Figure 3b. Furthermore, the RVE model is discretized with eight-noded, reduced-integrated, hexahedral elements and contains 8000 elements as well as 9261 nodes. During the simulation, the simulation based on the macro-scale model is conducted first, and then the boundary conditions of the micro-scale model are taken from the deformation histories of the macro-scale model.

## 3. Constitutive Models and Parameter Calibration

Here, the crystal plasticity finite element model and the Chaboche model are coupled with the same low-cycle fatigue damage model. The former is labeled as the CPFEM–LCFD model, and the latter is the Chaboche–LCFD model. Moreover, the Chaboche–LCFD and CPFEM–LCFD models are applied to the macro-scale model and the micro-scale model, respectively. These two models are described in this section.

### 3.1. Brief Introduction to the Chaboche–LCFD Model

The strain equivalence hypothesis [46] is employed here to describe the damage response ahead of the crack tip under overload. For this reason, the von Mises yield criterion, which accounts for kinematic hardening, fatigue damage, and isotropic hardening, is defined as follows:

(1) $$f=\sqrt{\frac{3}{2}\left(S/\left(1-d\right)-X\right):\left(S/\left(1-d\right)-X\right)}-R-\sigma_{0}=0,$$

where d is the damage factor; S is the deviatoric part of the Cauchy stress tensor; X is the back stress tensor; *R* is the isotropic hardening variable; and $\sigma_{0}$ is the yield stress at the moment when no plastic strain is generated [47]. The back stress tensor X is decomposed into three parts [48,49]:

(2) $$X=\sum_{j=1}^{3}X_{j}.$$

The evolution of the component $X_{j}$ is defined as follows [48,49]:

(3) $${\dot{X}}_{j}=\frac{2}{3}C_{j}\varepsilon^{p}-\lambda_{j}X_{j}\dot{p},$$

where $\varepsilon^{p}$ is the plastic strain tensor; $C_{j}$ and $\lambda_{j}$ (*j* = 1, 2, 3) are material constants; and $\dot{p}$ is the accumulated plastic strain rate.

The variable *R* in Equation (1) is calculated using the following equation [48,49]:

(4) $$\dot{R}=l\left[Q_{\mathrm{sat}}-R\right]\dot{p},$$

where l is the material constant, and $Q_{\mathrm{sat}}$ is the value representing the saturated strain hardening. Under the condition that the initial value R=0, Equation (4) is integrated and then becomes the following expression [49]:

(5) $$R=Q_{\mathrm{sat}}\left(1-\exp\left(-l\cdot p\right)\right).$$

### 3.2. Brief Introduction to the CPFEM–LCFD Model

To describe the elastic and plastic deformation, the deformation gradient **F** is introduced and is defined as follows:

(6) $$F=F^{e}F^{p},$$

in which $F^{e}$ is the elastic deformation gradient and $F^{p}$ is the plastic deformation gradient. Furthermore, the plastic velocity gradient $L^{p}$ is introduced to describe plastic deformation and is defined as follows [50]:

(7) $$L^{p}=\sum_{\alpha =1}^{\zeta }{\dot{\gamma }}^{\alpha }s^{\alpha }⊗m^{\alpha }.$$

in which *ζ* is the number of major slip systems; ${\dot{\gamma }}^{\alpha }$ is the shear strain rate on the *α*th slip system; $s^{\alpha }$ is the unit vectors of slip direction; and $m^{\alpha }$ is the unit vectors of slip plane normal.

Schmid’s law shows that dislocation motion will take place on the slip system if the resolved shear stress is not less than the critical value [51]. The resolved shear stress can be calculated as follows:

(8) $$\tau^{\alpha }=\sigma :P^{\alpha },$$

where $\tau^{\alpha }$ is resolved shear stress on the *α*th slip system, and **σ** is the Cauchy stress tensor.

The effective stress $\tilde{\sigma }$ is defined as follows [46]:

(9) $$\tilde{\sigma }=\frac{\sigma }{1-d}.$$

Thus, the effective resolved shear stress is expressed as follows:

(10) $${\tilde{\tau }}^{\alpha }=\tilde{\sigma }:P^{\alpha }=\frac{\tau^{\alpha }}{1-d}.$$

The slipping rate is defined as follows:

(11) $${\dot{\gamma }}^{\alpha }={\langle \frac{\left|\tau^{\alpha }/\left(1-d\right)-\chi^{\alpha }\right|-Q^{\alpha }}{K}\rangle }^{n}sign\left(\frac{\tau^{\alpha }}{1-d}-\chi^{\alpha }\right),$$

where $\langle \cdot \rangle$ is the Macaulay bracket; *n* is the strain rate sensitivity parameter; *K* is the positive viscosity parameter; $\chi^{\alpha }$ is the back stress on the *α*th slip system; and $Q^{\alpha }$ is the threshold stress. $\chi^{\alpha }$ is expressed as follows [52]:

(12) $${\dot{\chi }}^{\alpha }=r_{1}{\dot{\gamma }}^{\alpha }-r_{2}\chi^{\alpha }\left|{\dot{\gamma }}^{\alpha }\right|,$$

where $r_{1}$ and $r_{2}$ are the material constants. Considering the effect of the forest and debris dislocation densities on the threshold stress, $Q^{\alpha }$ in Equation (11) is thus expressed as follows [53]:

(13) $$Q^{\alpha }=\tau_{0}+\tau_{\mathrm{for}}^{\alpha }+\tau_{\mathrm{deb}},$$

in which $\tau_{0}$ is the initial threshold stress; $\tau_{\mathrm{for}}^{\alpha }$ and $\tau_{\mathrm{deb}}$ are the stresses caused by the forest dislocations on the *α*th slip system and the debris dislocations, respectively. $\tau_{\mathrm{for}}^{\alpha }$ and $\tau_{\mathrm{deb}}$ are provided by the following equations [53]:

(14) $$\tau_{\mathrm{for}}^{\alpha }=b\mu G\sqrt{\sum_{\beta =1}^{\zeta }L^{\alpha \beta }\rho_{\mathrm{tot}}^{\beta }},$$

(15) $$\tau_{\mathrm{deb}}=k_{\mathrm{deb}}Gb\sqrt{\rho_{\mathrm{deb}}}\log\left(\frac{1}{b\sqrt{\rho_{\mathrm{deb}}}}\right),$$

where *G* is the shear modulus; *b* is the value of the Burgers vector; *μ* is the interaction constant; $k_{\mathrm{deb}}$ is the material constant; $\rho_{\mathrm{deb}}$ is the debris dislocation density, $\rho_{\mathrm{tot}}^{\beta }$ is the total forest dislocation density on the *β*th slip system, and the initial dislocation density is set as 1 × 10^9^ m^−2^ according to the study by Pesin et al. [54]. $L^{\alpha \beta }$ is the latent hardening interaction matrix, for which all coefficients are shown in Table 1 for FCC material [55]. It can be seen from Table 1 that the number of independent coefficients is six. Table 2 shows these six independent coefficients.

**Figure 4:**
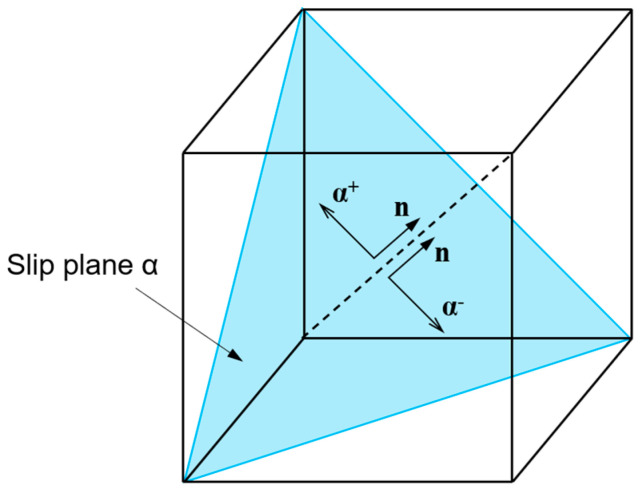
Schematic illustration of splitting slip system *α* into two systems $\alpha^{+}$ and $\alpha^{-}$.

Each slip system includes the reversible and irreversible dislocations. The irreversible dislocations depend on the strain path and will be annihilated if the strain path is reversed [56]. As a result, each slip system can be split into two systems. For example, the *α*th slip system has the systems $\alpha^{+}$ and $\alpha^{-}$, as shown in Figure 4. It can be seen that the slip directions of these two systems are opposite. Thereby, the total forest dislocation density can be decomposed into three parts:

(16) $$\rho_{\mathrm{tot}}^{\alpha }=\rho_{\mathrm{faw}}^{\alpha }+\rho_{\mathrm{rev}}^{\alpha^{+}}+\rho_{\mathrm{rev}}^{\alpha^{-}},$$

where $\rho_{\mathrm{tot}}^{\alpha }$ is the total forest dislocation density on the *α*th slip system; $\rho_{\mathrm{faw}}^{\alpha }$ is the density of irreversible forest dislocation; $\rho_{\mathrm{rev}}^{\alpha^{+}}$ is the density of the reversible forest dislocation on the system $\alpha^{+}$; and $\rho_{\mathrm{rev}}^{\alpha^{-}}$ is the density of the reversible forest dislocation on the system $\alpha^{-}$. $\rho_{\mathrm{rev}}^{\alpha^{+}}$ and $\rho_{\mathrm{rev}}^{\alpha^{-}}$ are expressed as follows [53]:

(17) $$\frac{\partial \rho_{\mathrm{faw}}^{\alpha }}{\partial \gamma^{\alpha }}=\left(1-P\right)k_{1}\sqrt{\sum_{\beta =1}^{\zeta }H^{\alpha \beta }\rho_{\mathrm{tot}}^{\beta }}-k_{2}\left(\dot{\varepsilon },T\right)\rho_{\mathrm{for}}^{\alpha },$$

$$\left(\mathrm{if}\;d\gamma^{\alpha^{+}}>0\right)$$

(18) $$\frac{\partial \rho_{\mathrm{rev}}^{\alpha^{+}}}{\partial \gamma^{\alpha }}=Pk_{1}\sqrt{\sum_{\beta =1}^{\zeta }H^{\alpha \beta }\rho_{\mathrm{tot}}^{\beta }}-k_{2}\left(\dot{\varepsilon },T\right)\rho_{\mathrm{rev}}^{\alpha^{+}},$$

(19) $$\frac{\partial \rho_{\mathrm{rev}}^{\alpha^{-}}}{\partial \gamma^{\alpha }}=-k_{1}\sqrt{\sum_{\beta =1}^{\zeta }H^{\alpha \beta }\rho_{\mathrm{tot}}^{\beta }}{\left(\frac{\rho_{\mathrm{rev}}^{\alpha^{-}}}{\rho_{0}^{\alpha }}\right)}^{m},$$

$$\left(\mathrm{if}\;d\gamma^{\alpha^{+}}<0\right)$$

(20) $$\frac{\partial \rho_{\mathrm{rev}}^{\alpha^{+}}}{\partial \gamma^{\alpha }}=-k_{1}\sqrt{\sum_{\beta =1}^{\zeta }H^{\alpha \beta }\rho_{\mathrm{tot}}^{\beta }}{\left(\frac{\rho_{\mathrm{rev}}^{\alpha^{+}}}{\rho_{0}^{\alpha }}\right)}^{m},$$

(21) $$\frac{\partial \rho_{\mathrm{rev}}^{\alpha^{-}}}{\partial \gamma^{\alpha }}=Pk_{1}\sqrt{\sum_{\beta =1}^{\zeta }H^{\alpha \beta }\rho_{\mathrm{tot}}^{\beta }}-k_{2}\left(\dot{\varepsilon },T\right)\rho_{\mathrm{rev}}^{\alpha^{-}},$$

where *P* is the reversibility factor; $H^{\alpha \beta }$ is the interaction matrix, the coefficients of which are all set to 1.0 [53]; $\rho_{0}^{\alpha }$ is the total dislocation density at the moment when the shear strain is generated in the opposite direction; $k_{1}$ is the statistically stored dislocation generation rate; and $k_{2}$ is the parameter in relation to the dynamic recovery and is defined as follows [57]:

(22) $$k_{2}=k_{1}\frac{b\mu }{\psi }\left[1-\frac{kT}{\hat{D}b^{3}}\ln\left(\frac{\dot{\gamma }}{{\dot{\gamma }}_{0}}\right)\right],$$

where *T* represents the absolute temperature; *ψ* is the effective activation energy; *k* denotes the Boltzmann’s constant; ${\dot{\gamma }}_{0}$ is the reference strain rate; and $\hat{D}$ is the drag stress.

The evolution of debris dislocation density is provided by the following equation:

(23) $$\Delta \rho_{\mathrm{deb}}=\sum_{\alpha =1}^{\zeta }q^{\alpha }b\sqrt{\rho_{\mathrm{deb}}}\frac{\partial \rho_{\mathrm{rem},\mathrm{for}}^{\alpha }}{\partial \gamma^{\alpha }}\left|\Delta \gamma^{\alpha }\right|,$$

where $q^{\alpha }$ is the parameter associated with the dislocation recovery rate.

### 3.3. Damage Evolution Model

Plastic deformation is generated in the vicinity of the crack tip, where the low cycle fatigue generally takes place [58,59,60]. To describe the damage response under overload, utilizing the low-cycle fatigue model is essential. A low-cycle fatigue damage evolution model proposed by Lemaitre is expressed as follows [61]:

(24) $$\dot{d}={\left(\frac{-Y}{S_{0}}\right)}^{s_{0}}\frac{\langle {\dot{\sigma }}_{\mathrm{eq}}\rangle }{\frac{\omega }{M}{\langle \frac{\sigma_{\mathrm{eq}}-\sigma_{Y}\left(1-d\right)}{\omega }\rangle }^{1-M}-\sigma_{Y}{\left(\frac{-Y}{S_{0}}\right)}^{s_{0}}},$$

where $\sigma_{\mathrm{eq}}$ is the von Mises equivalent stress; *ω*, *M*, *S*_0_, and *s*_0_ are the material constants; *Y* denotes the rate of damage strain energy release; $\sigma_{Y}$ is the yield stress. The damage strain energy release rate *Y* is defined as follows:

(25) $$Y=-\frac{1}{2}C:\varepsilon^{e}:\varepsilon^{e},$$

where $\varepsilon^{e}$ is the elastic strain tensor, and **C** is the tensor of elastic coefficients. The research by Lemaitre [61] shows that $\sigma_{Y}{\left(\frac{-Y}{S_{0}}\right)}^{s_{0}}$ in Equation (24) can be neglected, and $s_{0}$ is set to 1. As a result, Equation (24) can become the following expression:

(26) $$\dot{d}=M\upsilon \left(\frac{-Y}{S_{0}}\right){\langle \sigma_{eq}-\sigma_{Y}\left(1-d\right)\rangle }^{M-1}\langle {\dot{\sigma }}_{eq}\rangle ,$$

where υ is the material constant. The stress state is reflected by stress triaxiality in combination with the Lode parameter, and both stress triaxiality and the Lode parameter influence damage evolution [62,63]. For this reason, *S*_0_ in Equation (26) is replaced with the expression $\vartheta /\left[3\left|\eta \right|+0.72\left(1-\xi^{2}\right)\right]$ in light of our previous study [64], and then Equation (26) becomes the following expression:

(27) $$\begin{array}{cc} \dot{d} & =M\upsilon \left(\frac{-Y}{\vartheta /\left[3\left|\eta \right|+0.72\left(1-\xi^{2}\right)\right]}\right){\langle \sigma_{eq}-\sigma_{Y}\left(1-d\right)\rangle }^{M-1}\langle {\dot{\sigma }}_{eq}\rangle \\ & =M\upsilon \left(\frac{-Y}{\vartheta }\right)\left[3\left|\eta \right|+0.72\left(1-\xi^{2}\right)\right]{\langle \sigma_{eq}-\sigma_{Y}\left(1-d\right)\rangle }^{M-1}\langle {\dot{\sigma }}_{eq}\rangle \end{array}$$

in which *ϑ* is the material constant; *ξ* is the Lode parameter; and *η* is stress triaxiality.

### 3.4. Parameter Calibration

Both the Chaboche–LCFD and CPFEM–LCFD models contain elastic parameters and parameters related to low-cycle fatigue damage, strain hardening, and the flow rule. Here, all parameters in the CPFEM–LCFD model are extracted from our previous work [60]. For the Chaboche–LCFD model, the three elastic parameters *C*_11_, *C*_12_, and *C*_44_ are given as follows [65]:

(28) $$\begin{array}{l} C_{11}=\frac{E\left(1-\bar{\upsilon }\right)}{\left(1+\bar{\upsilon }\right)\left(1-2\bar{\upsilon }\right)}\\ C_{12}=\frac{E\bar{\upsilon }}{\left(1+\bar{\upsilon }\right)\left(1-2\bar{\upsilon }\right)},\\ C_{44}=\frac{\left(C_{11}-C_{12}\right)}{2} \end{array}$$

where *E* is the elastic moduli and is set to 71,000 MPa according to the research by Xu [66]; $\bar{\upsilon }$ is the Poisson’s ratio and is set to 0.31. Based on Equation (28) and the values of the elastic moduli and the Poisson ratio, three elastic parameters *C*_11_, *C*_12_, and *C*_44_ can be determined.

By assuming the damage during a fatigue cycle is constant, Equation (27) is integrated as follows:

(29) $$\frac{\partial d}{\partial N}=4\int_{\left(1-d\right)\sigma_{Y}}^{\sigma_{\max}}\frac{MR\sigma^{2}\left[3\left|\eta \right|+0.72\left(1-\xi^{2}\right)\right]{\langle \sigma -\left(1-d\right)\sigma_{Y}\rangle }^{M-1}}{2E\vartheta {\left(1-d\right)}^{2}}d\sigma ,$$

where *N* denotes the number of cycles.

There is no damage before fatigue loading, and the damage factor *d* reaches the critical value at failure. Under this condition, Equation (29) becomes the following expression via integration:

(30) $$\frac{d_{cr}}{2\left[\left(M^{2}+M\right)\sigma^{2}+2M\sigma \sigma_{Y}+2\sigma_{Y}^{2}\right]{\left(\sigma -\sigma_{Y}\right)}^{M}}=\frac{2MR\left[3\left|\eta \right|+0.72\left(1-\xi^{2}\right)\right]N_{f}}{E\vartheta \left(M^{3}+3M^{2}+2M\right)},$$

where *N_f_* is the number of cycles when failure takes place; $\left|\eta \right|=1/3$ and $\xi^{2}=1$ under the condition of uniaxial fatigue loading; and *d*_cr_ is the critical damage factor. Thus, the parameters *M*, *R*, and *ϑ* in Equation (30) can be determined based on three stress amplitudes and the corresponding fatigue lives. Here, the damage parameters of the Chaboche–LCFD model are extracted from our previous study [64].

The other parameters of the Chaboche–LCFD model are set as follows. Firstly, the stable hysteresis loop simulated at a given strain amplitude is obtained based on the trial values of these undetermined parameters. Then, the simulated stable hysteresis loop is compared with the measured data, and these parameters are fine turned until a good match between them is obtained. Finally, the stable hysteresis loop simulated at another strain amplitude should be compared with the corresponding measured data to validate the parameters. In this process, the simulation is conducted by using the RVE model with periodic boundary conditions. The parameters in the Chaboche-LCFD model are provided in Table 3, and those in the CPFEM-LCFD model are shown in Table 4. The comparison between the stable hysteresis loop simulated based on these parameters and the measured data is discussed in the following.

The RVE model with periodic boundary conditions is used to conduct the simulation at a strain amplitude of 1.0% for parameter calibration. The strain ratio is set to −1. Due to the fact that the material is continuous and non-overlapping, the periodic boundary conditions [67] are used in this section.

**Figure 5:**
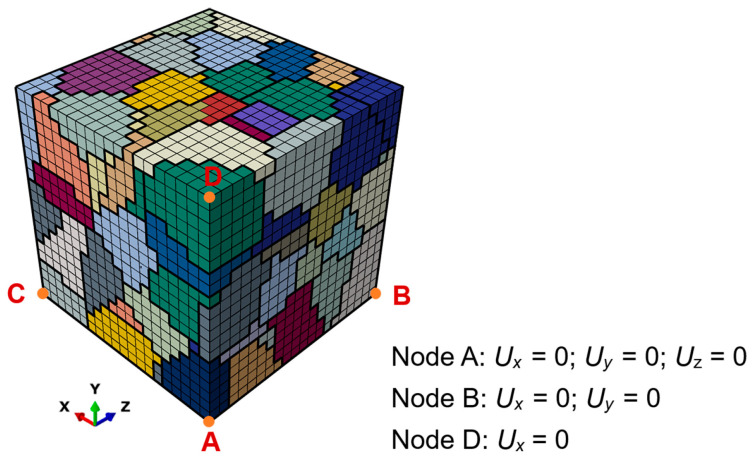
RVE model with periodic boundary conditions for parameter calibration.

Figure 5 demonstrates the RVE model with periodic boundary conditions. Node A is fixed in the *x*, *y*, and *z* directions; node B is fixed in the both the *x* and *y* directions; and node D is fixed in the *x* direction [68]. The external load is imposed on node D. Furthermore, the displacements of the nodes on the positive and negative *X*-planes are labeled as $u^{X+}$ and $u^{X-}$, respectively, and the following relationship can be obtained under the periodic boundary conditions:

(31) $$u^{X+}-u^{X-}=\varepsilon_{ij}u^{\mathrm{node}C},$$

where $u^{nodeC}$ is the displacement of node C, and $\varepsilon_{ij}$ denotes the strain. For the nodes on the *Y*- and *Z*-planes, their relationships are formulated as follows:

(32) $$u^{Y+}-u^{Y-}=\varepsilon_{ij}u^{\mathrm{node}D},$$

(33) $$u^{Z+}-u^{Z-}=\varepsilon_{ij}u^{\mathrm{node}B},$$

in which $u^{nodeB}$ and $u^{nodeD}$ are the displacements of node B and node D, respectively.

**Figure 6:**
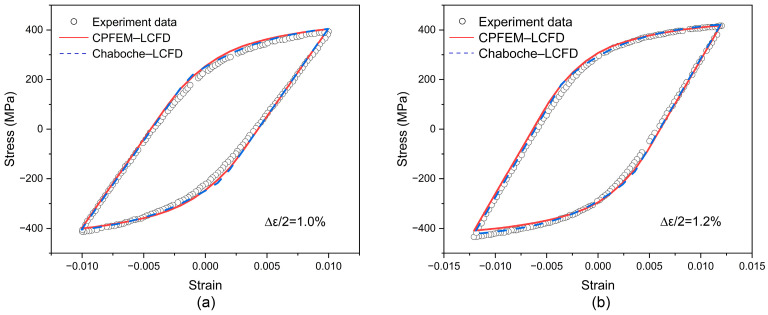
Measured and simulated stress–strain data of the Chaboche–LCFD and CPFEM–LCFD models at different strain amplitudes: (**a**) 1.0%; (**b**) 1.2%.

Figure 6a displays the measured and simulated cyclic stress–strain data at a strain amplitude of 1.0%. A good agreement between them is obtained. To validate the calibrated parameters, the simulation is also conducted at the strain amplitude of 1.2%, and the measured and simulated cyclic stress–strain data at the strain amplitude of 1.2% are shown in Figure 6b. The measured stress–strain data are consistent with those reported by Xu [66], and the measured data can also be found in Appendix A. Figure 6a,b indicate that both models with the calibrated parameters are capable of characterizing the mechanical response of a 7075 aluminum alloy.

## 4. Discussion

### 4.1. Evolution of Dislocation Density and Fatigue Damage Ahead of Crack Tip

**Figure 7:**
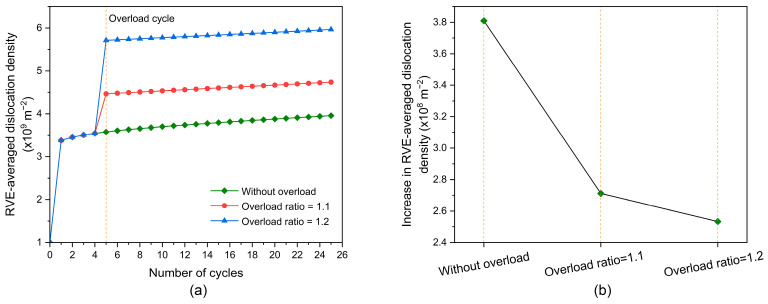
(**a**) Evolution of RVE-averaged dislocation density with increasing fatigue cycles; (**b**) increase in RVE-averaged dislocation density between the 6th and 25th cycles.

As shown in Figure 2, the overload condition is applied at the fifth cycle. The overload ratios of 1, 1.1, and 1.2 are used in this section. Overloading does not occur under the condition of *R*_OL_ = 1. At these three overload ratios, the evolution of RVE-averaged dislocation density with increasing fatigue cycles is shown in Figure 7a. At the overload ratios of 1.1 and 1.2, the dislocation density significantly increases at the fifth cycle; however, the growth of dislocation density slows down after the fifth cycle. Figure 7b shows the increase in RVE-averaged dislocation density between the 6th and 25th cycles. The case without overload has the largest increase in RVE-averaged dislocation density between the 6th and 25th cycles, followed by the case of *R*_OL_ = 1.1 and then the case of *R*_OL_ = 1.2. This indicates that the overload effect tends to decrease the growth of dislocation density before the dislocation density saturates. Plastic deformation causes the dislocation multiplication that increases dislocation density [69,70], and plastic deformation occurs if the slip system is active [71]. Consequently, activated slip systems are closely correlated with the dislocation density evolution after overload.

**Figure 8:**
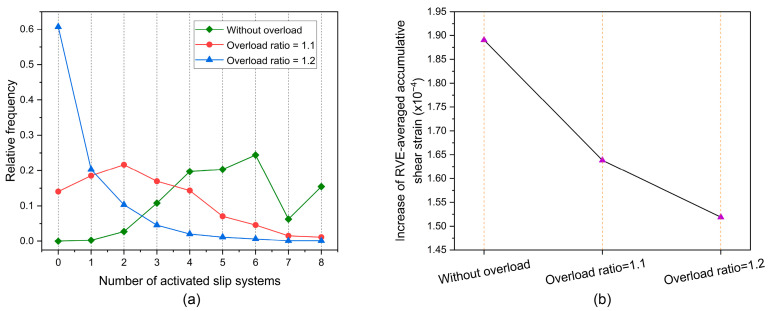
(**a**) Frequency distribution of slip systems activated at the peak of the 25th cycle; (**b**) increase in RVE-averaged accumulative shear strain at the 25th cycle.

Figure 8a shows the frequency distribution of the number of activated slip systems at the peak of the 25th cycle. The overload effect reduces the number of activated slip systems. Figure 8b shows the increase in RVE-averaged accumulative shear strain at the 25th cycle. It can be seen that the overload effect also reduces the increase in RVE-averaged accumulative shear strain. This indicates that the increase in dislocation density lowers accordingly. From a comparison of Figure 8a,b, it is reasonable to argue that for grains ahead of the crack tip, the reduced number of activated slip systems lowers the rate of increase in dislocation density after overload.

**Figure 9:**
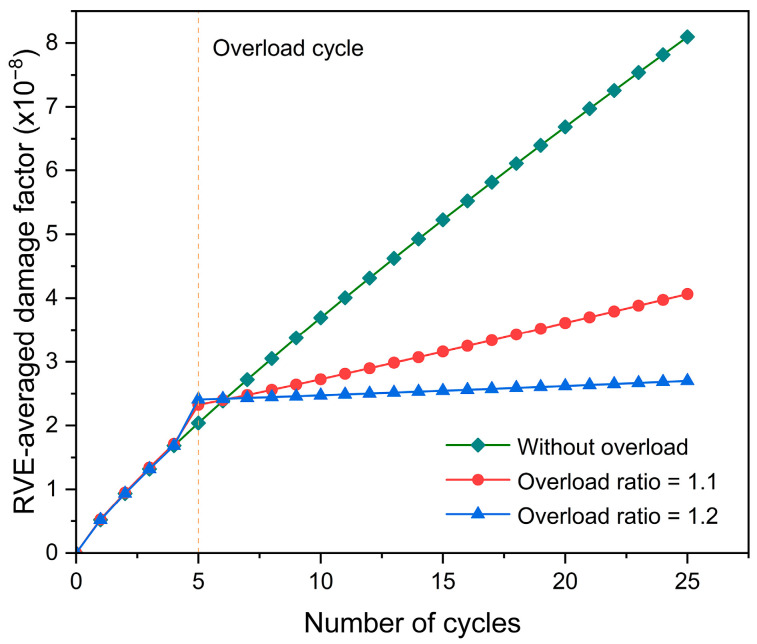
Evolution of RVE-averaged damage factor with increasing fatigue cycles.

Figure 9 displays the evolution of the RVE-averaged damage factor with increasing fatigue cycles. The overload effect reduces the rate of increase in damage. Combined with the dislocation density evolution shown in Figure 7a, it is extrapolated the rate of increase in dislocation density is associated with the damage evolution and can be considered as an indicator of the damage increase rate under the condition of cyclic loading with overload.

**Figure 10:**
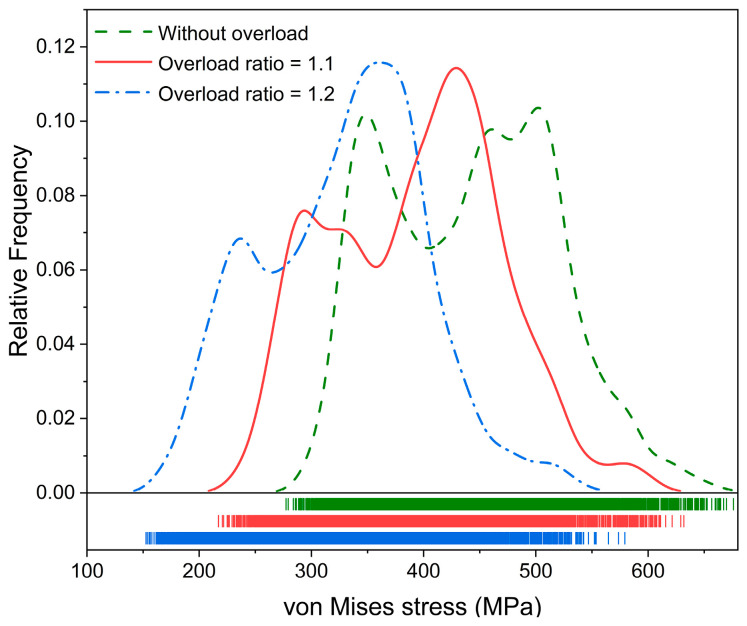
Distribution of the von Mises stress under conditions of different overload ratios at the peak of the 25th cycle.

The von Mises stress of each element within the RVE model was calculated and extracted to establish the distribution diagram, which is displayed in Figure 10. The distribution curve moves to the left with an increase in the overload ratio, indicating that the von Mises stress tends to decrease because of the overload effect. The compressive residual stress is generated ahead of the crack tip after overload. The magnitude of the stress at the crack can be reduced due to the presence of the compressive residual stress [34]. Furthermore, higher overloading generally leads to higher compressive residual stress [27]. Therefore, the decrease in von Mises stress is attributed to the compressive residual stress. The resolved shear stress on the slip system decreases once the von Mises stress is reduced after overload, which is the reason for the decreases in the number of activated slip systems and the increased shear strain.

In conclusion, the overload effect reduces the number of activated slip systems ahead of the crack tip, causing a higher rate of increase in fatigue damage accumulation and dislocation density.

### 4.2. Effect of Crystallographic Orientation on Damage Accumulation Ahead of Crack Tip

**Figure 11:**
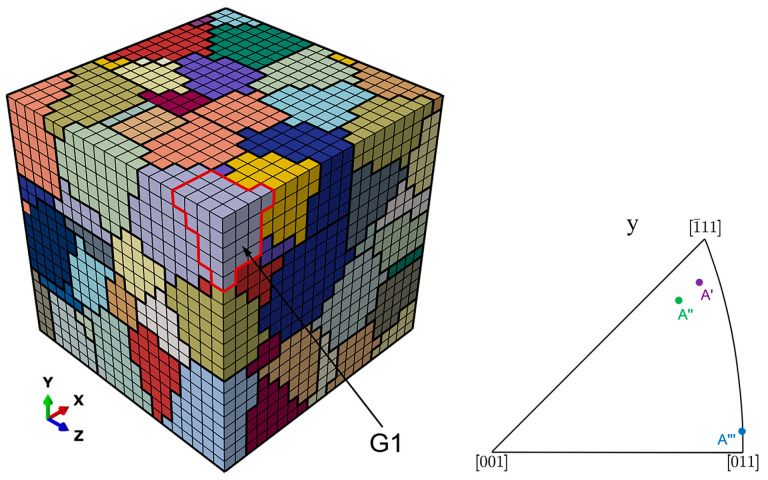
Position of grain G1 and inverse pole figure for three orientations.

To investigate whether crystallographic orientation affects the damage evolution under overload for the grain ahead of the crack tip, the grain labeled as G1, the boundaries of which are highlighted in red in Figure 11, is assigned three different orientations described by three Euler angles: orientation A′ (18.99, 37.63, 21.26), orientation A″ (198.52, 34.01, 37.4), and orientation A‴ (31.44, 4.09, 13.23). The inverse pole figure in the *y* direction for these three crystallographic orientations is also displayed in Figure 11.

**Figure 12:**
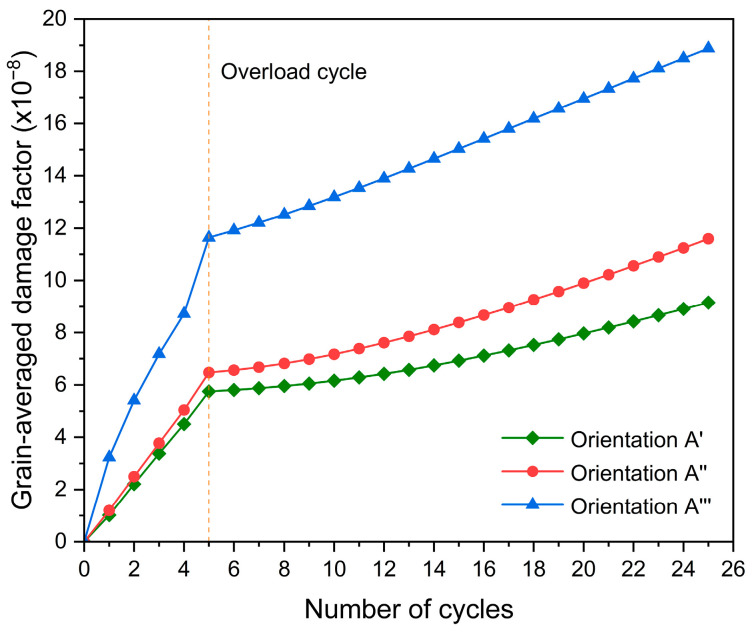
Grain-averaged damage factor evolution of grain G1 with increasing fatigue cycles.

Figure 12 shows the grain-averaged damage factor of grain G1 that increases with increasing fatigue cycles. For grain G1, its damage evolution depends on its crystallographic orientation. Particularly, the rates of increase in damage are all found to decrease after overload for the three crystallographic orientations, confirming that the fatigue crack growth rate can be decreased by the overload effect. Plastic slip results in the accumulation of fatigue damage in metals [72]. As a result, the effect of orientation on the damage accumulation relies on plastic slip on the slip planes under overload.

**Figure 13:**
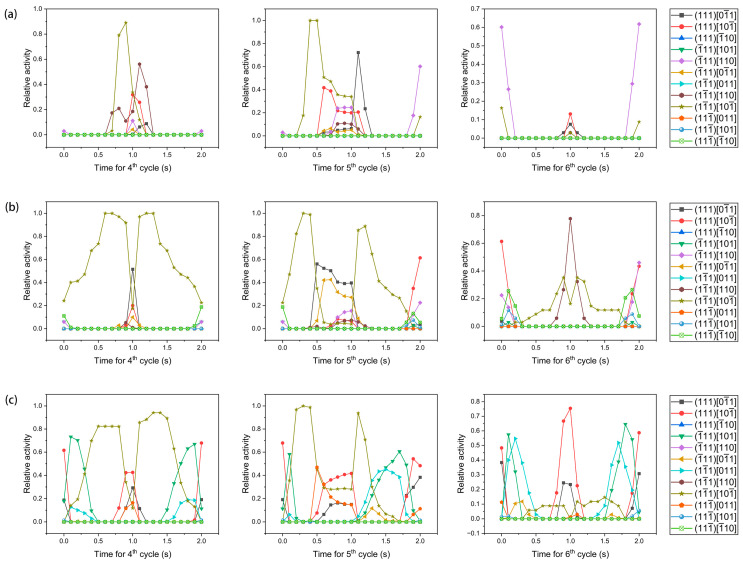
Relative activities of 12 slip systems at the fourth, fifth, and sixth cycles under different orientations: (**a**) orientation A′, (**b**) orientation A″, and (**c**) orientation A‴.

The relative activities of 12 slip systems for grain G1 at the fourth, fifth and sixth cycles are shown in Figure 13, where the relative activity $\bar{r}$ is defined by the following equation [73]:

(34) $${\bar{r}}^{\alpha }=\frac{1}{V}\int r^{\alpha }dv,$$

in which *r^α^* is the ratio of the shear strain generated on the *α*th slip system to the total shear strain of the 12 slip systems at a given point in time for a material point; *dv* is the volume of a material point; *V* is the total volume of the grain. Figure 13 indicates that for grain G1, its activated slip systems depend on its crystallographic orientation. In addition, it can also be found that the activated slip systems before overload are different from those after overload.

**Figure 14:**
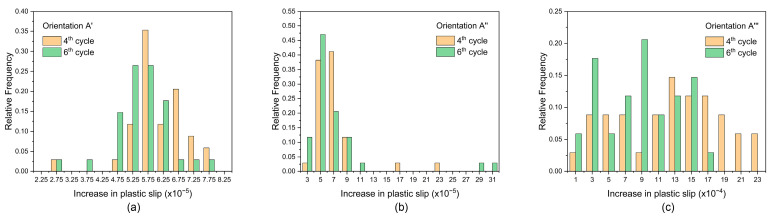
Frequency distribution of increase in plastic slip at the fourth and sixth cycles for (**a**) orientation A′, (**b**) orientation A″, and (**c**) orientation A‴.

For each element within grain G1, the increases in plastic slip at the fourth and sixth cycles are calculated and extracted to establish the histograms that are shown in Figure 14. For each orientation, the increase in plastic slip at the sixth cycle is less than that at the fourth cycle, implying that the rate of increase in damage reduces. In combination with the variation in slip systems dominating the plastic slip shown in Figure 13, it is plausible to consider that the variation in slip system activity caused by the overload effect contributes to the fact that the rate of increase in damage lowers after overload. In addition, there is a difference in the increase in plastic slip at the fourth or sixth cycle for each orientation, implying that the rate of increase in damage is different for each orientation. This indicates the influence of crystallographic orientation on damage.

### 4.3. Effect of the Nearest Neighbor Grains on Damage Evolution Ahead of Crack Tip

Under the cyclic load of constant amplitude, the interaction between the nearest neighbor grains has an influence on the damage accumulation of the grain [41]. Similarly, it can be extrapolated that the damage evolution of the grain is also affected by the crystallographic orientations of its nearest neighbor grains under overload. To validate this deduction, the damage evolution of the target grain, of which the nearest neighbor grains are respectively assigned different sets of crystallographic orientations, is described in this section.

**Figure 15:**
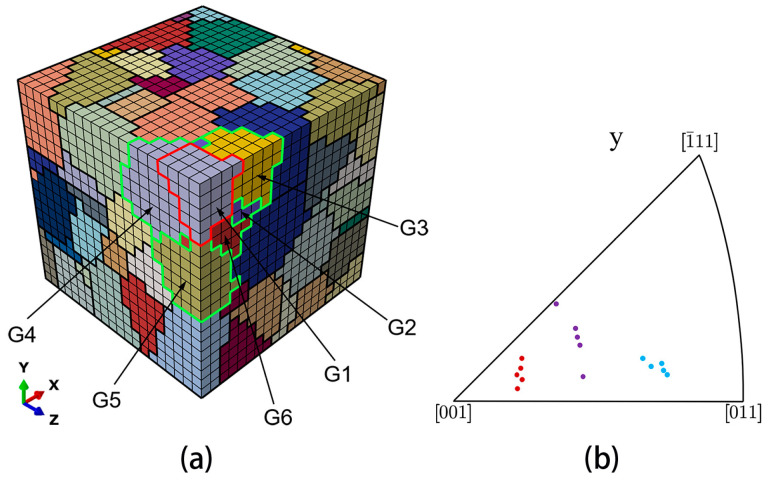
(**a**) Schematic diagram of the nearest neighbor grains of grain G1; (**b**) inverse pole figure in the *y* direction for Set B′ (blue), Set B″ (purple), and Set B‴ (red).

Here, grain G1 (mentioned in Section 4.2) was selected to be the target grain. Grain G1 and its five nearest neighbor grains are shown in Figure 15a, where the boundaries of the nearest neighbor grains (G2~G6) are highlighted in green. The crystallographic orientations of the nearest neighbor grains are divided into three sets: Set B′, Set B″, and Set B‴, and their distribution is shown in Figure 15b. Set B′ is identified by the blue color in Figure 15b, Set B″ by the purple color and Set B‴ by the red color. As shown in Table 5, for each individual grain of Set B′, Set B″, and Set B‴, the Schmid factors are 0.36, 0.39, and 0.42, respectively. Moreover, the misorientation between grain G1 and each individual neighbor grain is 35°.

**Figure 16:**
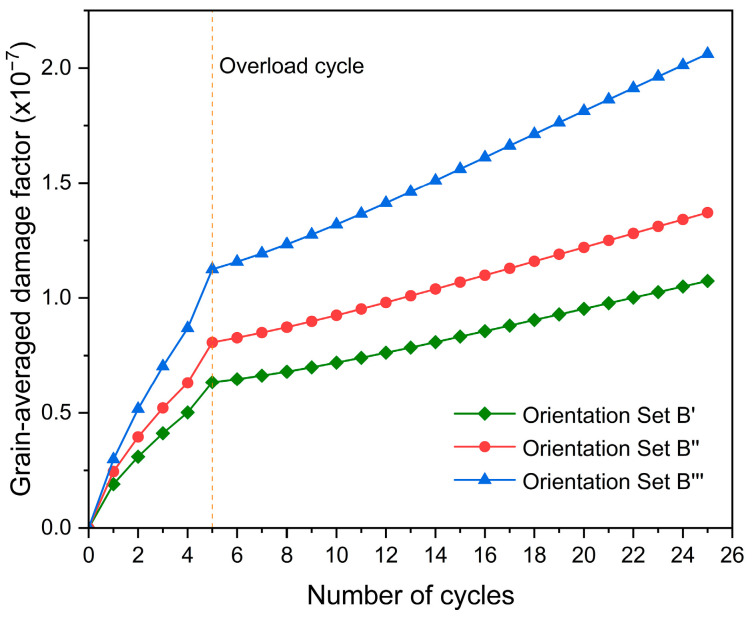
Evolution of the grain-averaged damage factor of grain G1 with increasing fatigue cycles.

The evolution of the grain-averaged damage factor for grain G1 with increasing fatigue cycles, when surrounded by different sets of the nearest neighbor grains, is shown in Figure 16. Set B‴ has the largest damage values during the cyclic loading, followed by Set B″, and then Set B′, indicating that the damage evolution of grain G1 is significantly influenced by the orientations of the nearest neighbor grains. Moreover, it should be noted that the rates of increase in damage all decrease after overload for three sets of the nearest neighbor grains.

**Figure 17:**
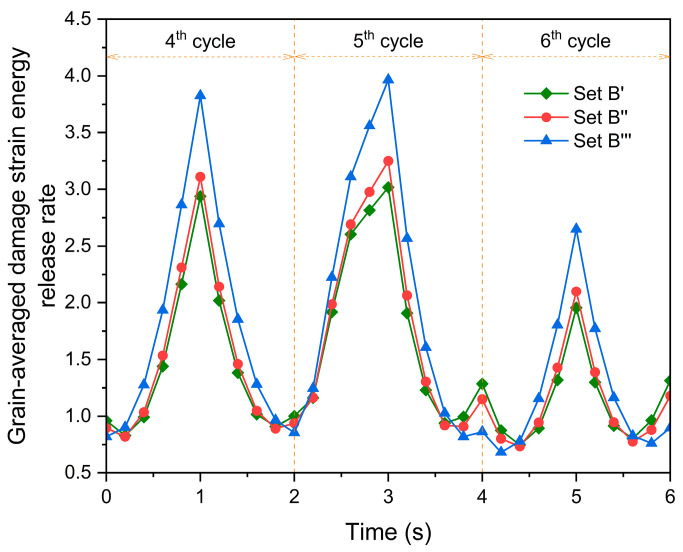
Evolution of the grain-averaged damage strain energy release rate for grain G1 during the fourth, fifth, and sixth cycles.

**Figure 18:**
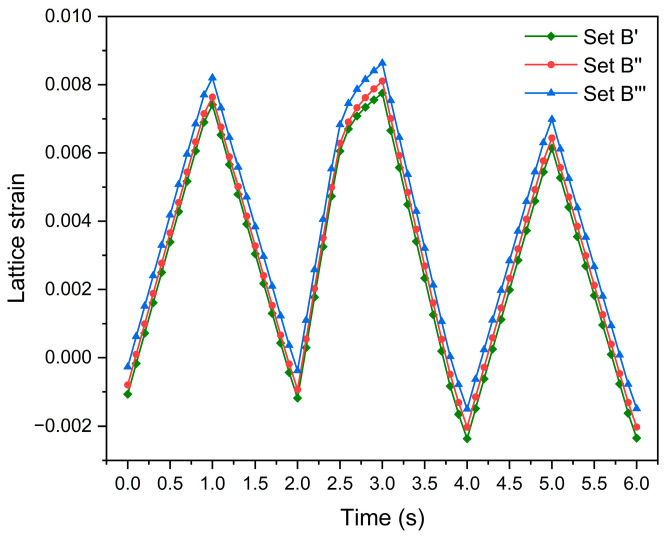
The {1 1 0} lattice strain evolution of grain G1 during the fourth, fifth, and sixth cycles.

Figure 17 shows the evolution of the grain-averaged damage strain energy release rate of grain G1 at the fourth, fifth, and sixth cycles, and indicates that the evolution of the damage strain energy release rate relies on the orientations of the nearest neighbor grains. This is attributed to the different local constraints prescribed by the three sets of the nearest neighbor grains. The damage strain energy release rate, taken into account by Equation (27), governs damage accumulation [46]. The comparison of Figure 17 and Figure 18 indicates that for grain G1, the damage strain energy release rate is a factor affecting its damage evolution. The damage strain energy release rate is caused by the combined action of stress and elastic strain. Consequently, it is considered that the nearest neighbor grains influence both the stress level and elastic strain of grain G1, ultimately affecting its rate of increase in damage.

### 4.4. Effect of the Nearest Neighbor Grains on Lattice Strain Evolution Ahead of Crack Tip

As mentioned above, elastic strain affects the damage strain energy release rate. Here, the lattice strain is taken as a measure of elastic strain to characterize the elastic strain variation caused by overload. The lattice strain evolution of the target grain, which is affected by the crystallographic orientations of the nearest neighbor grains, is studied in this section. As in Section 4.3, grain G1 and its nearest neighbor grains are selected.

The lattice strain is calculated using the following expression [74]:

(35) $$\varepsilon^{hkl}=\iota_{i}\varepsilon_{ij}^{elastic}\iota_{j},$$

where $\varepsilon^{hkl}$ is the {*h k l*} lattice strain, ι is the vector of reflection direction, and $\varepsilon^{elastic}$ is the tensor of elastic strain.

Figure 18 shows the evolution of the {1 1 0} lattice strain for grain G1 in the *y* direction during the fourth, fifth and sixth cycles. The {1 1 0} lattice strain of grain G1 evolves differently under different orientations of the nearest neighbor grains. This can be explained by the deformation mismatch between grain G1 and its nearest neighbor grains and indicates the influence of the crystallographic orientations of the nearest neighbor grains. Furthermore, the magnitude of lattice strain at the sixth cycle is smaller than the corresponding magnitude at the fourth cycle for each case, indicating that the overload effect reduces the magnitude of the lattice strain.

**Figure 19:**
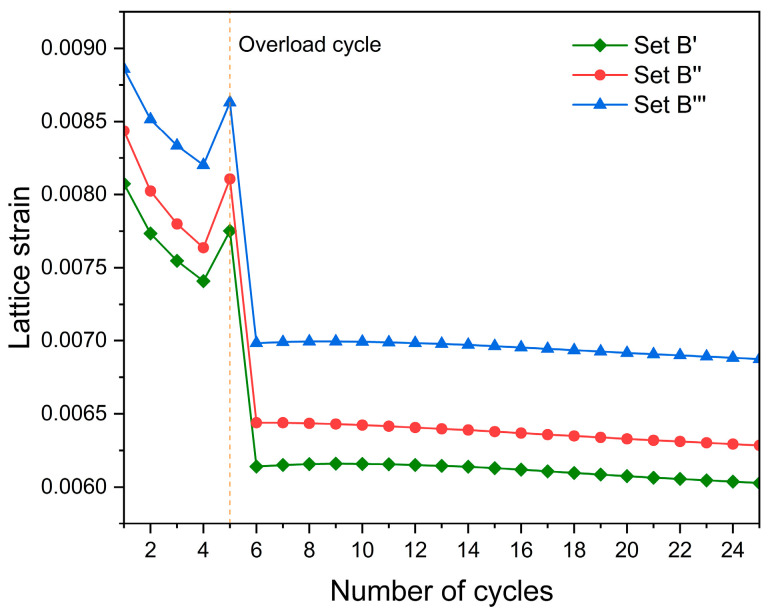
The {1 1 0} lattice strain evolution of grain G1 at the peaks of 25 cycles.

Figure 19 shows that as the number of fatigue cycles increases, the {1 1 0} lattice strain tends to gradually decrease. This result, which is similar to the conclusion of Zheng et al. [42], shows that the residual lattice strain gradually vanishes as the damage continues to accumulate.

## 5. Conclusions

Based on the crystal plasticity model coupled with the low-cycle fatigue damage effect, the retardation mechanisms of a 7075 aluminum alloy were investigated in this study from a microscopic perspective. The microstructure-dependent fatigue response ahead of the crack tip was discussed. The following main conclusions regarding the response of grains ahead of the crack tip under overload were drawn:

(1) The number of activated slip systems is reduced because of the overload effect, causing the rate of increase in dislocation density and damage to decrease before the dislocation density is saturated.
(2) The overload effect changes the crystallographic-orientation-dependent activities of the slip systems, which contributes to the fact that the rate of increase in damage lowers after overload.
(3) The stress and elastic strain of the grain are affected by the crystallographic orientations of its nearest neighbor grains, which eventually affects the rate of increase in damage.
(4) The lattice strain of the grain decreases and is significantly affected by the crystallographic orientations of its nearest neighbor grains.

## Figures and Tables

**Table 1:** Coefficients of the matrix *L^αβ^*. AA, BB, CC and DD are the slip planes $(\overset{-}{1}11)$, (111), $(11\overset{-}{1})$, and $(1\overset{-}{1}1)$, respectively. The index numbers 1 to 6 denote the slip directions [011], $[0\overset{-}{1}1]$, [101], $[\overset{-}{1}01]$, $[\overset{-}{1}10]$, and [110], respectively.

	AA_2_	AA_3_	AA_6_	BB_2_	BB_4_	BB_5_	CC_1_	CC_3_	CC_5_	DD_1_	DD_4_	DD_6_
AA_2_	*M*_0_	*M*_1_	*M*_1_	*M*_3_	*M*_4_	*M*_4_	*M*_2_	*M*_4_	*M*_5_	*M*_2_	*M*_5_	*M*_4_
AA_3_		*M*_0_	*M*_1_	*M*_4_	*M*_2_	*M*_5_	*M*_4_	*M*_3_	*M*_4_	*M*_5_	*M*_2_	*M_4_*
AA_6_			*M*_0_	*M*_5_	*M*_5_	*M*_2_	*M*_5_	*M*_4_	*M*_2_	*M*_4_	*M*_4_	*M*_3_
BB_2_				*M*_0_	*M*_1_	*M*_1_	*M*_2_	*M*_5_	*M*_4_	*M*_2_	*M*_4_	*M*_5_
BB_4_					*M*_0_	*M*_1_	*M*_5_	*M*_2_	*M*_4_	*M*_4_	*M*_3_	*M*_4_
BB_5_						*M*_0_	*M*_4_	*M*_4_	*M*_3_	*M*_5_	*M*_4_	*M*_2_
CC_1_							*M*_0_	*M*_1_	*M*_1_	*M*_3_	*M*_4_	*M*_4_
CC_3_								*M*_0_	*M*_1_	*M*_4_	*M*_2_	*M*_5_
CC_5_									*M*_0_	*M*_4_	*M*_5_	*M*_2_
DD_1_										*M*_0_	*M*_1_	*M*_1_
DD_4_											*M*_0_	*M*_1_
DD_6_												*M*_0_

**Table 2:** Six independent coefficients of the matrix *L^αβ^* [53].

*M*_1_	*M*_2_	*M*_3_	*M*_4_	*M*_5_	*M*_6_
0.068	0.068	0.0454	0.625	0.137	0.122

**Table 3:** Parameters for the Chaboche–LCFD model.

*C*_1_	*λ*_1_	*C*_2_	*λ*_2_	*C*_3_
30,000 MPa	350	3000 MPa	70	265 MPa
*λ*_3_	*σ*_0_	*Q*_sat_	l	
50	255 MPa	55 MPa	15	

**Table 4:** Parameters for the CPFEM–LCFD model.

*C*_11_	*C*_12_	*C*_44_	*K*	*n*	*r*_1_
98,413 MPa	44,214 MPa	27,099 MPa	35 MPa	50	25,000 MPa
*r*_2_	*τ*_0_	*μ*	*b*	*G*	*k*_deb_
650	95 MPa	0.9	2.86 × 10^−10^ m	27,862 MPa	0.086
*k*_1_	*ψ*	$\hat{D}$	${\dot{\gamma }}_{0}$	*q*	*p*
5 × 10^6^ m^−1^	0.55	75.61 MPa	10^7^	685 MPa	0.8
*σ*_Y_	*ϑ*	*υ*	*M*		
405 MPa	35.5	3.5 × 10^−13^	2.2		

**Table 5:** Misorientation and Schmid factors of grains.

Misorientation	Schmid Factor			
	Grain G1	Set B′	Set B″	Set B‴
35°	0.33	0.36	0.39	0.42

**Figure A1:**
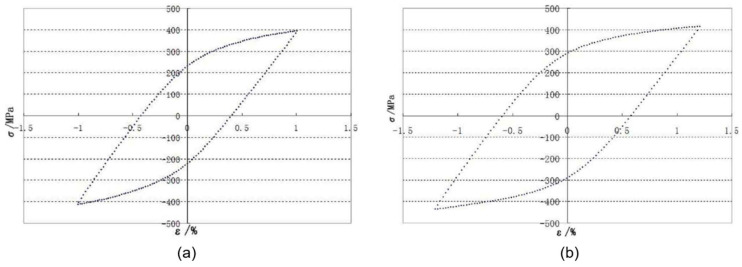
Measured stress–strain data at different strain amplitudes: (**a**) 1.0%; (**b**) 1.2%.

## Data Availability

The raw data supporting the conclusions of this article will be made available by the authors on request.

## Appendix A

The measured stress–strain data at the strain amplitudes of 1.0% and 1.2%, which are extracted from reference [66], are shown as follows:
